# Teleradiology, the Internet, and the development of multidisciplinary professional networks: new times for the specialty?

**DOI:** 10.1590/0100-3984.2017.50.3e1

**Published:** 2017

**Authors:** Luiz Felipe Nobre

**Affiliations:** 1 PhD, Full Member of the Colégio Brasileiro de Radiologia e Diagnóstico por Imagem (CBR), Adjunct Professor of Radiology at the Universidade Federal de Santa Catarina, Medical Director at netPACS Tecnologia, São José, SC, Brazil. E-mail: luizfelipenobresc@gmail.com.

While thinking about the aspects that I consider relevant to the positive impact that teleradiology applications on the Internet have and the opportunities that they offer for better practice in our specialty, I decided to begin researching the phenomenon of social networks, a contemporary context into which we are all inserted, not only as doctors but also as citizens, in an increasingly interconnected world.

In his excellent lecture "The Revolution of the New"^(1)^, the philosopher Leandro Karnal expounded upon the question: Do social networks distance people from each other or bring them closer together? He introduces his reasoning by referring to the danger that exists in the conservative position often hidden within a questioning of the problematic "new", which is compared with an idealized past that is far from reality.

Seeking to bring this discussion to our professional activity, in which we emphasize the need to "return to our roots", attempting to elicit a more humane position and expand the role of the radiologist in medical interventions, we criticized teleradiology severely, characterizing it as a milieu in which doctors would no longer engage with patients, hiding behind technological tools, offering services that are impersonal, and devaluing those services, while creating an uneven playing field for "local" physicians.

In my estimation, that is exactly where the danger lies! In traditional practice, even though we know that our reports often represent inconclusive evaluations, accompanied by outdated documentation, with printouts (still!) of the images in the original plane of acquisition, without any type of link within the text, are we actually offering quality? Does sending the hundreds or even thousands of original images on media such as CDs achieve the goal of adequately clarifying the findings to the requesting physician and thus confer value on the work of the radiologist?

I firmly believe that we must avoid a Manichean evaluation, broadening our view of the potential benefits that the new web-based techniques can offer us. We have now, as never before, the opportunity to be more available, reaching an ever-increasing number of patients and requesting physicians, offering them results that are more objective, clarifying, and easily accessible, with the possibility of discussing cases even at a distance, through chat or videoconference-allowing analysis of the images in real time-available on conventional computers, tablets, and smartphones, without the need to install new programs.

New PACS, operating in a distributed network, have allowed the visualization of images and the creation of online reports, facilitating the organization of work processes and greatly favoring group report and review activities, regardless of the distance between observers. As well as making the results available electronically to patients and requesting physicians in an innovative format (such as reports including hyperlinked text and images), via the Internet, these new tools have transformed the practice of many radiology clinics around the world, allowing them to provide results in a more timely manner and to involve a multidisciplinary team, facilitating access to the radiologist and, consequently, adding enormous value to our work.

As occurs in social networks, the presence of radiologists in multidisciplinary professional online networks has the transforming potential of a great window of visibility for our specialty, breathing new life into the traditional concept of teleradiology. The same tool that allows an unscrupulous professional to work almost anonymously, when used well, can bring the radiologist back into the limelight.
